# Benefits in Cardiac Function from a Remote Exercise Program in Children with Obesity

**DOI:** 10.3390/ijerph20021544

**Published:** 2023-01-14

**Authors:** Savina Mannarino, Sara Santacesaria, Irene Raso, Massimo Garbin, Andreana Pipolo, Silvia Ghiglia, Gabriele Tarallo, Annalisa De Silvestri, Matteo Vandoni, Daniela Lucini, Vittoria Carnevale Pellino, Giuseppina Bernardelli, Alessandro Gatti, Virginia Rossi, Valeria Calcaterra, Gianvincenzo Zuccotti

**Affiliations:** 1 Pediatric Cardiology Unit, Pediatric Department, Buzzi Children’s Hospital, 20154 Milan, Italy; 2 Biometry & Clinical Epidemiology, Scientific Direction, Fondazione IRCCS Policlinico San Matteo, 27100 Pavia, Italy; 3 Laboratory of Adapted Motor Activity (LAMA), Department of Public Health, Experimental Medicine and Forensic Science, University of Pavia, 27100 Pavia, Italy; 4 BIOMETRA Department, University of Milan, 20129 Milan, Italy; 5 Exercise Medicine Unit, Istituto Auxologico Italiano, IRCCS, 20135 Milan, Italy; 6 Department of Industrial Engineering, University of Rome Tor Vergata, 00133 Rome, Italy; 7 DISCCO Department, University of Milan, 20122 Milan, Italy; 8 Pediatric Unit, Pediatric Department, Buzzi Children’s Hospital, 20154 Milan, Italy; 9 Department of Internal Medicine, University of Pavia, 27100 Pavia, Italy; 10 Department of Biomedical and Clinical Science, University of Milano, 20157 Milan, Italy

**Keywords:** obesity, cardiac function, cardiac morphology, children, adolescents, physical activity, remote exercise, cardiovascular prevention

## Abstract

Physical activity (PA) is a crucial factor in preventing and treating obesity and related complications. In this one-arm pre–post longitudinal prospective study, we evaluated the effects of a 12-week online supervised training program on cardiac morphology, function and blood pressure (BP) in children with obesity. The training program consisted of three sessions per week, each lasting 60 min. Advanced echocardiographic imaging (tissue Doppler and longitudinal strain analysis) was used to detect subclinical changes in heart function. Categorical variables were described as counts and percentages; quantitative variables as the mean and standard deviation (SD) as they were normally distributed (Shapiro–Wilks test). Pre–post comparisons were made with a paired *t*-test. A total of 27/38 (71%) enrolled patients (18M/9F, 11 ± 2 years) completed the training protocol and were considered in the analysis. At baseline, no hypertensive patient was noted; all echocardiographic variables were within the normal range. After training, we observed a significant reduction in BP parameters, including systolic BP values and Z-score, diastolic BP values, centiles and Z-score, and mean arterial pressure (all *p* < 0.05). Significant variations in echocardiographic interventricular septum (IVSd) thickness (*p* = 0.011), IVSd Z-score (*p* = 0.001), left ventricular (LV) end-diastolic diameter (*p* = 0.045), LV posterior wall thickness Z-score (*p* = 0.017), and LV global longitudinal strain (*p* = 0.016) were detected. No differences in LV diastolic function and right ventricular strain were noted. PA plays a decisive role in improving BP control and has benefits on left ventricle systolic function, representing a strategic approach to limit CV risk. Online exercise could be an excellent method of training in children with obesity.

## 1. Introduction

The increasing incidence of obesity has become a significant public health concern [1]. The World Health Organization (WHO) estimated that more than 39 million children under the age of 5 years old are overweight or obese [2]. This epidemy is widespread due to the interactions between multiple factors, including genetic, biological, developmental, behavioral and cultural issues [3].

Obesity in childhood affects multiple organ systems in an adverse manner, causing insulin resistance, dyslipidemia, type 2 diabetes mellitus (T2DM), metabolic syndrome, non-alcoholic fatty liver disease (NAFLD), excess stress on the musculoskeletal system [4,5,6], pulmonary disorders (obstructive sleep apnea, asthma and chronic obstructive pulmonary disease) related to direct mechanical changes due to fat deposition, decreased pulmonary volume, restricted diaphragmatic mobility and rib movement [7,8], pro-inflammatory state, and psychological and social problems [9].

Furthermore, obesity has a great impact on the cardiovascular system [10,11]; the body mass index (BMI) in children and adolescents has been shown to have a positive correlation with the risk of heart disease in adults, suggesting that cardiovascular damage may begin during childhood [12]. The excessive adiposity increases the metabolic request of the body, resulting in an increased blood volume and preload of the heart [13]. Moreover, the increased peripheral vasoconstriction and renal tubular sodium reabsorption, together with the augmented sympathetic activity and the overactivation of the renin–angiotensin system, contribute to vascular alterations such as increased arterial stiffness and resistance [14]. Those alterations lead to an augmented afterload of the heart, to both concentric and eccentric hypertrophy and to the obesity-related hypertension with arterial wall damage [15]. Furthermore, the cardiac structure can be modified by being overweight: research in children with obesity demonstrated a positive relationship between BMI and cardiac dimensions and an increased left ventricular (LV) mass compared with normal weight children [16,17,18]. Altered morphology has been associated with impaired cardiac function. Diastolic dysfunction has been reported in children with obesity and strain and strain rate analyses have shown contractile abnormalities even within those with a normal LV ejection fraction [19,20]. A reduction in longitudinal strain is a sensitive measure and a predictor of cardiovascular disease and mortality [21].

Despite how widespread this disease is, interventions for the prevention and management of obesity in children are still not well defined and difficult to assess [22] Physical activity (PA) is a crucial factor in the fight against overweight and the literature agrees on advocating exercise as among the most useful tools for both the prevention and treatment of obesity and related complications [23]. Among PA benefits, there are the increase in muscular strength, the improvement in endothelial function, the reduction in insulin resistance and lipid profile, the reverse of cardiovascular impairment and the reduction in blood pressure [24,25,26]. Moreover, exercise was demonstrated to improve the cardiac function measured as global longitudinal strain in adults and children with cardiovascular disease [21,27]. However, PA can also be difficult to practice for children with obesity due to psychological problems related to body shame and social pressure; but no studies have been made to test the feasibility of a program of online exercise.

The aim of this study was to evaluate the effects of a program of remote PA on echocardiographic and blood pressure (BP) parameters in children with obesity. The use of advanced echocardiographic imaging, such as tissue Doppler and longitudinal strain analysis, has been made to detect subclinical changes in heart function. The hypothesis is that online exercise could improve cardiac function and arterial pressure, regardless of weight loss. PA training in remote modalities may also represent a useful tool to prevent early cardiovascular risk [21,23,28].

## 2. Material and Methods

### 2.1. Patients

This is a one-arm pre–post longitudinal prospective study performed at the Pediatric Department of the Children’s Hospital Vittore Buzzi of Milan with the collaboration of Laboratory of Adapted Motor Activity (LAMA), University of Pavia, Italy. A total of thirty-eight children with obesity, referred to our pediatric unit for obesity by their primary care pediatric consultant, were consecutively enrolled (March 2021–November 2021) in this study. Obesity was defined as a BMI z-score ≥ 2 according to the World Health Organization [2]. Patients suffering from chronic illnesses, taking medications, with contraindications to the practice of PA or whose obesity was due to a secondary condition were excluded. Twenty-seven patients accomplished the training protocol and completed cardiologic follow up. To better understand the exclusive effect of PA on blood pressure (BP) and cardiac function, no personalized calorie restriction regimen was provided during the observation period in our study.

### 2.2. Methods

#### 2.2.1. Auxological Evaluation

In all the participants, height, weight, puberal stage, BMI, BMI z-score, waist circumference (WC), and waist-to-height ratio (WHtR) were measured as previously described [29]. Briefly, weight was measured using a scale platform (Seca, Hamburg, Germany) with children not wearing shoes and in light clothing, standing upright, hands at their sides, and looking straight ahead. Height was measured using a Harpenden stadiometer (Holtain Ltd., Cross-well, UK) [29]. BMI was calculated as body weight (kilograms) divided by height (meters squared) and converted into BMI z scores using WHO reference values [30]. WC was measured in the horizontal plane midway between the lowest ribs and the iliac crest, using a flexible inch tape [29]. Waist to height ratio (WHtR) was calculated according to Maffeis [31]. Puberal stages were considered according to Tanner and classified as prepuberal stage 1 = Tanner 1; middle puberty stage 2 = Tanner 2–3; late puberty stage 3 = Tanner 4–5 [29].

#### 2.2.2. Cardiologic Assessment

Cardiological evaluation with BP and echocardiography was performed before (T0) and within one week after the completion of the physical fitness protocol (T1). All children underwent standard and advanced transthoracic echocardiography (TTE), recorded with Vivid S5 GE Healthcare by a single pediatric cardiologist (M.G.) and validated by a second cardiologist (S.S). Standard echocardiographic measurements were made according to the American Society of Echocardiography Guidelines [32], including M-mode of the left ventricle (LV), diastolic LV function with mitral inflow peak velocities and tissue doppler images (TDI) of septal and lateral annular peaks and LV mass indexed for height [33]. Z-score indexed for body surface area were calculated according to the Detroit z-score [34]. The advanced TTE analysis included measures of myocardial deformation. Analysis was performed offline with independent software (TOMTEC Imaging Systems GmbH). Global longitudinal strain (GLS) was calculated as the average of the peak systolic longitudinal strain from all LV segments and for the RV function, the peak longitudinal strain of the free wall (RVFWLS) was measured from a 4-chamber image. According to published vendor normal range, we considered age-dependent normal values with a 95% confidence interval: 2–9 years old −21.7% (95% CI, −23.0% to −20.5%), 10–13 years old from −20% (95% CI, −20.8% to −19.1%) and 14–21 years old −19.9% (95%CI, −20.6% to −19.2%) [35]. If the values were within the normal range, a change in at least 10% from baseline in LVGLS was considered a significant variation. Peak global left atrial reservoir strain (LAS) was recorded from the 4-chamber view, as a diastolic function parameter.

BP was detected using multiple office blood pressure measurement (MOBP): systolic and diastolic values were the result of the arithmetic mean of ten successive measurements. Percentiles and z-scores [36] of the collected values were identified to investigate any variation after the intervention.

#### 2.2.3. Physical Fitness (PF) Tests

To evaluate PF in children, we used common field test batteries. Prior to and after the training protocol period, we assessed different domains of PF: cardiovascular fitness through the 6 min walking test performed in accordance with international guidelines [37,38], lower-limb power and strength through the standing broad jump [39,40] and speed-agility assessed through the 5 × 10 sprint test [41]. All procedures are described elsewhere [37,40,41,42,43,44]. During the test and training execution, we assessed the children’s effort with the Children’s Effort Rating Table (CERT) [45] to ensure the safety of the protocol. All the PF measurements were taken by two sport specialists after a two-week period of familiarization.

#### 2.2.4. Training Protocol

Children performed the training protocol for 12 weeks with three 60 min online sessions (on Mondays, Wednesdays and Fridays) per week, totaling 36 sessions that were supervised by two expert trainers. According to the literature, a 12-week protocol is sufficient to improve both physical fitness and health outcomes in children with obesity, reducing the risk of exercise program abandonment [46,47,48,49]. The training program was implemented from April to June 2021 and from September to December 2021. The exercise sessions were always performed from 5 PM to 6 PM, after school. Each session was streamed using the Zoom^®^ platform in real time, allowing live interaction among instructors and children. Moreover, three days per week, participants had to exercise individually using a YouTube channel (“LAMA Junior”) implemented with training video routines by the sport specialists. All the training sessions were structured with a 5–10 min warm-up, 20 min of aerobic interval training, 20 min of a strength circuit, and 5–10 min of cool-down or stretching [44]. All the routines were proposed through playful and recreative activities and did not require any specific equipment. An example of the exercises proposed are shown in a previous study of Vandoni et al. (2022) [44]. To understand the intensity of the training session, the heart rate during the session was monitored with an activity tracker (Fitbit Charge 2©, Fitbit Inc., San Francisco, CA, USA), to stay between 60 and 80% of their maximum heart rate to reach an intensity classifiable from moderate to vigorous and registered by the trainers after 30 min from the beginning of the sessions. Moreover, before each exercise, trainers reminded children to maintain a safety effort based (low–moderate intensity) on a CERT scale value, as described elsewhere [50].

### 2.3. Statistical Analysis

Categorical variables were described as counts and percentages; quantitative variables as the mean and standard deviation (SD) as they were normally distributed (Shapiro–Wilks test). Pre–post comparisons were made with a paired t-test. All analyses are performed with Stata v17.0 (StataCorp USA). Power consideration: with 27 subjects, it will be possible to achieve a power >90% to find a significant (*p* < 0.05) mean pre–post difference when SD is 1.5-fold greater than mean difference.

## 3. Results

In Table 1 are reported the baseline characteristics of the 27 patients with obesity (18 males, 9 females, aged 11 ± 2 years, range 8–15) who completed the training protocol (adherence to the exercise program of 90%).

All patients underwent a cardiac evaluation at baseline and within one week after the end of the PA program (T1).

An improvement was recorded between baseline and after the exercise program in WHtR without reaching statistical significance (*p* = 0.06); no significant differences were noted in BMI z-score (*p* = 0.38) and WC (*p* = 0.52). Puberal stage remained stable in all patients, except in two in which there was a progression from prepuberty to middle puberty (*p* = 0.19).

In our population, at baseline, no patient was hypertensive but 6/27 (22.2%) had high–normal systolic pressure before intervention (Z-score between 2 and 3) and 4/6 (66.7%) improved their Z-score to <2.

Table 2 presents variations in BP from baseline (T0) to after training control (T1). A statistically significant reduction in all the BP parameters was observed after intervention: systolic BP values (*p*= 0.05) and systolic BP Z-score (*p* = 0.027), diastolic BP values (*p*= 0.001), diastolic centiles (*p* <0.063) and diastolic BP Z-score (*p* <0.001) and mean arterial pressure (MAP) (*p*= 0.002).

Concerning the echocardiographic evaluation, at baseline, all the measured variables were within the range of normality for body surface area and age. We recorded only 6 subjects (22.2%) with a mildly increased left ventricular end-diastolic diameter (LVEDD) with a LVEDD z-score between 2 and 3. Both left ventricular diastolic and systolic function were normal at baseline and remained normal at the follow-up. Table 3 depicts variations in echocardiographic parameters at baseline and after training (T1).

A statistically significant variation was evident for interventricular septum thickness (IVSd) (*p* = 0.011) and IVSd z-score (*p*-value 0.001), for the left ventricular end-diastolic diameter (*p* = 0.045) and the left ventricular posterior wall thickness z-score (*p*-value 0.017). LV mass indexed for height values showed no significant differences between T0 and T1 (*p* = 0.39).

The advanced evaluation did not show differences in LV diastolic function and right ventricular strain. The left ventricular global longitudinal strain (LVGLS) was normal at baseline but improved significantly after the completion of the PA program (*p*-value 0.016). In 11/27 patients (40.7%), a 10% LVGLS improvement was noted after training compared to baseline (range 10–22%).

## 4. Discussion

^2^Childhood obesity is a multisystem disease that leads to several comorbidities, cardiovascular diseases, hypertension, T2DM, NAFLD, hyperlipidemia and other conditions associated with chronic inflammation, which cause disability and shorten the life span [51]. Even if the effects of the increased cardiometabolic risk develop progressively, the combination of multiple risk factors certainly results in more severe consequences. Diet and PA combined is a useful non-pharmacological strategic intervention to reduce obesity-related complications. PA shows positive effects in reducing weight, improving insulin sensitivity, alleviating plasma dyslipidemia, normalizing BP, decreasing blood viscosity, reducing oxidative stress, improving leptin sensitivity and consequently protecting the heart and vessels [52,53,54] to reduce cardiovascular risk from childhood to adulthood [23,55].

This study was performed to evaluate the cardiovascular effects of remote PA in children with overweight and obesity, without providing a dietary regimen and therefore regardless of weight loss. Our study shows satisfactory participation in the project, with 71% of the children who completed the training program. Concerning the effect of exercise on BP, our analysis showed a significant improvement in all systolic, diastolic and MAP values of both absolute and BP z-score. Although there were no patients with hypertension in our population at baseline, 22% of them had high–normal systolic pressure before intervention, and 66.6% of them improved their Z-score. At the echocardiographic evaluation, normal chamber morphology and cardiac function at baseline was noted in all patients, indicating that obesity-related cardiac dysfunction was still not clinically evident in our population. Nevertheless, the echocardiographic assessment also showed a statistically significant reduction in IVSd and PWd thickness when considering concomitant child growth. In contrast, statistically significant increase in the absolute value of the LVEDD did not correspond to a significant Z-score increase, demonstrating that this parameter is likely to be dependent on patient growth. No significant changes occurred in parameters related to diastolic dysfunction and all patients had normal baseline global longitudinal strain, which further improved at T1; 41% of subjects showed a significant improvement in the LVGLS after the training program, considering a 10% change from baseline in LVGLS a significant variation [21,56]

A positive effect in multiple measures of muscular and cardiorespiratory fitness in children with overweight and obesity [57] is proven in team sports such as football [58] and recreational activities as well as active video games (“exergames”) [59]. However, less is known about the effect of the remote training program adapted to children with obesity. In our study, the high adherence to the PA program could be supported by the possibility to perform PA online, taking advantage of videos, exergames and personal training. Moreover, remote training offers numerous advantages in terms of feasibility, personal adaptation, cost effectiveness and psychological aspects; in fact, the children are in a familiar environment and freed from social pressures. Furthermore, PA increases parasympathetic activity, certainly influencing the autonomic system, arterial stiffness and endothelial function [11] in healthy subjects and patients with obesity. Our data confirm the positive effect of exercise on BP even when it is not conducted at high intensity and for a relatively short time. Moreover, BP changes affect left ventricular wall thickness. In our patients, the reduction in IVSd and PWd thickness after PA could be related to improvement in BP; however, no significant changes in LV cardiac mass were evident after the intervention. A possible explanation could be that the majority of patients had normal BP values at baseline, and thus did not show latent signs of cardiac mass modification in such a short time; further evaluation in a large population is needed for a better understanding. The absence of significant changes in diastolic function is probably due to the age of our population because impairment occurs later and requires a more severe cardiovascular compromise to manifest. On the other hand, the improvement in LV strain may suggest that PA and BP optimization bring functional advantage to LV, even between parameters in the normal range. It is hypothesized that another important role of physical activity on cardiac function and BP may depend on the effects of exercise on the autonomic nervous system. However, this hypothesis could not be analyzed in our study due to the low sample size.

It is well known that obesity can cause hypertension and that obesity and hypertension together can cause increased metabolic demand and cardiac remodeling, leading to high end-diastolic pressure and to diastolic dysfunction [60]. However, the effects of obesity on LV systolic function are less known with several studies which have reported a normal or even a supranormal ejection fraction in adult populations with obesity [61]. More advanced measurements, such as ventricular strain analysis, also showed subclinical myocardial dysfunction in those with a normal ejection fraction [19,20]. Our study results are in line with a recent metanalysis that Murray J. et al. [21] conducted on a large adult population: in that study, a significant change in LV strain was found after the physical exercise program lasting at least two weeks. Interestingly, they found a moderate effect on LVGLS only in the CV risk population and they found no effect in the healthy subgroup, and the effect of PA was independent to exercise intervention length. In the literature, data about the effect of PA on cardiac function in children are few and sometimes discordant. Obert P et al. [62] demonstrated that after a 13-week running program, cardiac dimension increased, and wall thickness decreased. In an old study, Hayashi et al. [63] reported a significant change in LV mass and dimension after 1 year of training in children with obesity. Those finding are in line with our data. However, another two studies (one in overweight children [27] and another in healthy children [64]) reported increased PWd thickness after training. The first study [27] was conducted among 20 children with obesity after 3 months of a football training intervention vs. a controlled group without a physical activity intervention. They demonstrated a relative reduction in BP in the intervention group compared to the control group, and no modifications in LVGLS [27]. However, Z-scores were not evaluated, and the type of PA is not comparable in the studies. Further research will help to better define the effect of PA on PWd thickness and left ventricle longitudinal myocardial deformation in childhood.

The main limitation of this study is the small number of participants; an increased sample size will be useful to validate these results, offering the possibility to also assess the effect of BP on cardiac function. Moreover, the effect of heart rate variability and autonomic activity could be not excluded; heart rate potentially reflects changes in sympathetic activity and may influence both BP values and cardiac function. Additionally, as adiposity indices, we considered only clinical parameters, which are inaccurate measures of body components; using a tool for the examination of body composition may be useful to evaluate the relationship between changes in fat and/or lean mass and echocardiographic assessment.

The findings of our study confirmed the importance of PA as a key treatment tool for improving health outcomes and preventing and reversing CV modifications in patients with obesity at any age. Online physical exercise is an interesting activity to be proposed for overweight children and could be implemented in schools or included as part of a medical program for the prevention and treatment of obesity.

## 5. Conclusions

Our study confirms that physical exercise plays a decisive role in improving BP and has positive effects on left ventricle systolic function, measured with advanced techniques. To prevent the progression of obesity from childhood to adulthood as well as the development of CV risks, it is necessary to teach children to exercise regularly. PA increases physical fitness and modifies children’s cardiometabolic risk even after a short period of training. Online exercise could be an excellent method of training for the pediatric population: it is appreciated by children and adolescents and could be more manageable for families.

## Figures and Tables

**Table 1:** Baseline data of the enrolled pediatric patients. Quantitative variables are expressed as the mean (±standard deviation, SD) and categorical variables are described as counts and percentages.

Baseline Characteristics	Time T0	Time T1
**Patient numbers (n)**	27	27
**Age (years)**	11.0 ± 2.0	11.2 ± 2.0
**Gender**		-
Male, n (%)	18 (67)	
Female, n (%)	9 (33)	
**Pubertal stages**		
Prepubertal, n (%)	16 (59.3)	14 (51.8)
Middle puberty, n (%)	5 (18.5)	7 (25.9)
Late puberty, n (%)	6 (22.2)	6 (22.2)
**Ethnicity**		-
White, non-Hispanic, n (%)	17 (63)	
Afroamerican, n (%)	4 (15)	
Hispanic or Latino, n (%)	6 (22)	
**Weight, kg**	65.57 ± 11.03	66.56 ± 10.89
**Weight, z-score**	1.97 ± 0.22	1.97 ± 0.52
**Height, cm**	149.9 ± 6.58	152.1 ± 4.95
**Height, z-score**	0.48 ± 1.2	0.59 ± 1.4
**Body mass index, kg/m^2^**	26.3 ± 0.6	26.0 ± 1.4
**Body mass index, z-score**	2.16 ± 0.5	2.03 ± 0.6
**Waist circumference, cm**	90.5 ± 11.2	88.6 ± 10.8
**Waist circumference/height ratio**	0.61 ± 0.05	0.58 ± 0.04

**Table 2:** Comparison between blood pressure measurements at baseline (T0) and after training (T1). Data are expressed as the mean (±standard deviation, SD). Z-scores are expressed as the mean (±SD).

Parameters	T0	T1	*p*-Value
**Systolic pressure** (mmHg)			
Mean (±SD)	117.0 ± 9.7	113.6 ± 10.3	0.05
Z-score, mean (±SD)	1.15 ± 0.84	0.82 ± 0.93	0.027
Centiles (±SD)	80.89 ± 17.82	72.44 ± 23.44	0.063
**Diastolic pressure** (mmHg)			
Mean (±SD)	69.3 ± 8.1	64.0 ± 7.0	0.001
Z-score, mean (±SD)	0.68 ± 0.67	0.16 ± 0.57	<0.001
Centiles (±SD)	70.07 ± 21.09	55.56 ± 20.31	0.001
**Mean arterial pressure** (mmHg)			
**Mean (±SD)**	85.2 ± 7.9	80.5 ± 7.4	0.002

**Table 3:** Comparison between the echocardiographic data at baseline (T0) and after training (T1). Data are expressed as the mean (±standard deviation, SD). Z-scores are expressed as the mean (±SD).

Echocardiographic Variables	T0 (n = 27)	T1 (n = 27)	*p*-Value
**RV systolic function**			
RVFWLS (%)	−23.9 (±2.1)	−23.7 (±2.4)	0.610
**LV systolic function**			
LV GLS (%)	−22.5 (±1.5)	−23.5 (±1.9)	0.016
**LV diastolic function**			
E/e’	5.79 (±0.78)	5.82 (±0.74)	0.856
LAS (%)	41.1 (±5.4)	41.1 (±4.93)	0.975
**LV measurements**			
EDD (mm)	42.5 (±3.7)	43.3 (±4.0)	0.045
EDD z-score	−1.09 (±1.05)	−1.01 (±1.06)	0.435
IVSd (mm)	7.84 (±0.96)	7.63 (±0.94)	0.011
IVSd z-score	0.19 (±0.48)	−0.001 (±0.41)	0.001
PWd (mm)	7.55 (±0.84)	7.47 (±0.82)	0.239
PWd z-score	0.41 (±0.59)	0.27 (±0.58)	0.017
LV mass indexed for height (g/m^2.7^)	33.61 (±6.35)	32.23 (±5.36)	0.393

RV (right ventricle), RVFWLS (right ventricle free wall longitudinal strain), LV (left ventricle), LV GLS (left ventricle global longitudinal strain), LAS (left atrium strain), EDD (end-diastolic diameter), IVSd (interventricular septum diastolic), and PWd (posterior wall diastolic).

## Data Availability

Not applicable.
